# A human vascularized microtumor model of patient-derived colorectal cancer recapitulates clinical disease

**DOI:** 10.1016/j.trsl.2022.11.011

**Published:** 2022-12-05

**Authors:** Stephanie J. Hachey, Agua Sobrino, John G. Lee, Mehraneh D. Jafari, Samuel J. Klempner, Eric J. Puttock, Robert A. Edwards, John S. Lowengrub, Marian L. Waterman, Jason A. Zell, Christopher C.W. Hughes

**Affiliations:** a Irvine Department of Molecular Biology and Biochemistry, University of California, Irvine, California; b Irvine School of Medicine, University of California, Irvine, California; c Department of Surgery, Weill Cornell Medicine, New York, New York; d Massachusetts General Hospital Cancer Center, Boston, Massachusetts; e Irvine Department of Mathematics, University of California, Irvine, California; f Irvine Department of Microbiology and Molecular Genetics, University of California, Irvine, California; g Irvine Department of Biomedical Engineering, University of California, Irvine, California

## Abstract

Accurately modeling tumor biology and testing novel therapies on patient-derived cells is critically important to developing therapeutic regimens personalized to a patient’s specific disease. The vascularized microtumor (VMT), or “tumor-on-a-chip,” is a physiologic preclinical cancer model that incorporates key features of the native human tumor microenvironment within a transparent microfluidic platform, allowing rapid drug screening *in vitro*. Herein we optimize methods for generating patient-derived VMT (pVMT) using fresh colorectal cancer (CRC) biopsies and surgical resections to test drug sensitivities at the individual patient level. In response to standard chemotherapy and TGF-*β*R1 inhibition, we observe heterogeneous responses between pVMT derived from 6 patient biopsies, with the pVMT recapitulating tumor growth, histological features, metabolic heterogeneity, and drug responses of actual CRC tumors. Our results suggest that a translational infrastructure providing rapid information from patient-derived tumor cells in the pVMT, as established in this study, will support efforts to improve patient outcomes.

The majority (~95%) of novel anticancer agents fail to progress through clinical trials despite showing promise during preclinical study, and those that do gain FDA-approval confer marginal survival gains at best.^1^ The low rates of effective preclinical compounds reaching the clinic can be largely attributed to weaknesses in current model systems, which turn out to be poor predictors of drug response in patients.^2^ Moreover, efforts to develop individualized treatment regimens that lead to durable responses have been hampered considerably by inter- and intrapatient tumor heterogeneity and the complexity of underlying biologic pathways.^3^ Precision medicine in oncology remains guided by somatic mutation assessment in isolation from the patient’s unique disease context,^4^ yielding heterogeneous responses to targeted therapies that underscore the need for predictive models that provide individualized information about drug response for each patient. Recent advancements in circulating tumor DNA (ctDNA) analyses are beginning to inform molecularly-based personalized treatment efforts^5^; however, limitations persist, including a short half-life of ctDNA in blood and low signal-to-noise ratio that can impede clinical evaluations.^5, 6^ Additionally, the utility of liquid biopsy in predicting response to novel therapies has yet to be realized.^6^ Establishing better tumor models, using patient-derived tissue, could thus improve both the drug pipeline, and also our ability to tailor treatment regimens to each individual patient. Hindering the development of this kind of approach is that primary cancer cells are surprisingly difficult to establish *in vitro*, limiting widespread use and study.^7, 8^ As a result, standard research practice has involved drug testing on established cell lines that, after maintenance in cell culture for years, are no longer representative of the disease from which they were derived.^9, 10^ Failure of preclinical models to mimic the patient-specific disease condition leads to the development and clinical testing of often weakly effective, and sometimes potentially harmful, drugs on patients. A new approach to personalized oncology is necessary to ensure that the most therapeutic benefit, with the least toxicity, is consistently achieved for each individual patient. To date, however, efforts to establish primary cancer models have shown variable success.^8, 11^ Furthermore, current model systems for drug screening, including organoid cultures^12^ and cancer cell monolayer cultures,^8^ fail to recapitulate the complex, heterogeneous 3-dimensional (3D) cell-cell interactions and microenvironment of a vascularized human tumor *in vivo*.

To address the need for improved preclinical models, we have validated a microfluidic device that supports the formation of a dynamic tumor ecosystem—the VMT.^13–15^ Via co-culture of multiple cell types in an extracellular matrix under dynamic flow conditions, a perfused vascular network forms *de novo* and feeds the growing tumor mass just as it does *in vivo*, with survival and growth of the tumor entirely dependent on flow of nutrients through the living vascular network. Importantly, the VMT contains only cells of human origin and can be established rapidly for turnaround of results in a clinically-actionable timeframe. We have previously shown that, in contrast to 2-dimensional (2D) and 3D monocultures (spheroids), the VMT reproduces the growth and drug sensitivity of *in vivo* tumors derived from 2 commonly used CRC cell lines, HCT116 and SW480.^15^ The VMT closely models the gene expression profiles seen when these tumors are grown *in vivo* as demonstrated by bulk and single-cell RNA sequencing, capturing clinically-relevant tumor heterogeneity not seen in 2D or 3D monocultures. Furthermore, the VMT captures drug responses dependent on tumor-stroma interactions, as seen by the effectiveness of the TGF-*β*R1 inhibitor galunisertib on SMAD4 mutant SW480 VMT growth, a result that is not seen in monolayer or spheroid cultures.^15^

The ability to directly grow and study samples from patient tumors promises to be a crucial technology in the clinical management of cancer and the development and testing of new therapies as part of the shift to personalized medicine in oncology. To this end, we optimized methods for generating patient-derived VMT (pVMT) using fresh CRC biopsies and surgical resections. Herein we show that the VMT model reproducibly supports the growth and long-term culture of patient-derived CRC cells, and that the pVMT accurately models tumor growth, metabolic heterogeneity and drug response seen in clinical specimens. We demonstrate the ability to interrogate *ex vivo* response to standard chemotherapy (FOLFOX—combination 5FU, oxaliplatin, and leucovorin) and TGF-*β*R1 inhibition using pVMTs derived from 4 primary CRC specimens and 2 PDX-derived primary cell lines. Primary CRC show heterogeneous drug sensitivities between patient samples, and we find that, compared to drug response in the VMT, spheroid cultures often overestimate therapeutic efficacy while missing galunisertib as a targeted therapeutic option in most instances. By comparing *in vitro* tumor growth and drug response with each patient’s actual clinical data, we find that the pVMT better recapitulates clinical CRC disease than standard spheroid culture.

## MATERIALS AND METHODS

### Sample collection

Tissue is collected under protocols approved by UCI IRB in accordance with the principles of ethical human subjects research. Written informed consent is obtained from all patients prior to enrollment in the study. The population is sampled from patients ≥18 years of age treated for CRC at UCI Medical Center (MC) in Orange, California. Willing and eligible patients with a known diagnosis of colon or rectal cancer who were already planned to undergo medically necessary endoscopic sampling or operative tumor resection were approached for participation in this study. Excess tissue not needed for diagnostic purposes was deposited directly into a conical tube with basal cell culture media on ice for immediate transport to UCI Main Campus for processing. All samples were de-identified at the time of collection. Laboratory personnel are trained annually in the safe handling of bloodborne pathogens and observe biosafety level III precautions while handling biohazardous materials.

### Tissue processing

Surgical specimens are cut into ~1 mm pieces using a scalpel and biopsies are broken into small pieces with pipetting. Tissue pieces are portioned for fixation with 4% PFA and cell culture. Portions reserved for cell culture are then resuspended in a 300 U/mL collagenase type III HBSS solution and digested at room temperature with vigorous shaking for 30 minutes at a time. Every 30 minutes, single cells and cell clusters are removed from the digestion, neutralized with medium and collected by centrifugation at 340 × *g* for 3 minutes. Cells were then resuspended into colorectal cancer initiating cell (CCIC) supplemented media and plated into ultra-low attachment plates. The procedure was repeated until all the tissue was fully digested.

### Cell culture

Tissues were transported in serum-free DMEM with 4.5 g/L glucose, sodium pyruvate and L-glutamine with 1× P/S and antimitotic/antimycotic. CCIC media consisted of DMEM F12 50:50 media with L-glutamine supplemented as indicated below (Table I).

The following were added freshly to basal CCIC media (Table II):

Endothelial growth medium-2 (EGM-2) was obtained from Lonza (Basel, Switzerland), DMEM was from Corning (Corning, NY), and Fetal Bovine Serum (FBS) was from Gemini Bio Products (Sacramento, CA). Endothelial colony forming cell-derived EC (ECFC-EC, hereby termed EC for endothelial cells) were isolated from human umbilical cords obtained from the UCIMC under an approved IRB protocol. Normal human lung fibroblasts (NHLF, hereby termed LF) were purchased from Lonza (Basel, Switzerland) and CCIC707 and CCIC1024 were gracious gifts from Dr Marian Waterman and Dr Steven Lipkin. EC were cultured in EGM-2 and used between passages 4–8. LF (used between passages 4–9). All cells were cultured at 37°C in a 5% CO_2_ incubator. Cells were washed with HBSS (Thermo Fisher Scientific), harvested using TrypLE express enzyme and collected by centrifugation at 340 × *g*, 22°C for 3 minutes. Primary tumor cells were fed with fresh CCIC media supplemented with 10 μM Rho kinase (ROCK) inhibitor Y-27683 every 2–3 days and expanded for no more than 7–10 days prior to device loading.

### Lentiviral transduction and cell labeling

The EC and cancer cells were transduced with lentivirus expressing mCherry (LeGO-C2 (plasmid #27339)) or green fluorescent protein (GFP) (LeGO-V2 (plasmid #27340), from Boris Fehse (Addgene, MA). In cases where transduction was not possible, cells were labeled with CFSE (Thermo Fisher Scientific) or cytoplasmic membrane dyes (Biotium) according to the manufacturer’s instructions.

### Flow cytometry

Cells were harvested from suspension culture and stained with anti-EpCAM FITC conjugated human antibody (CD326 clone REA764) or control FITC IgG (REA Control IgG S) at 1:50 dilution according to the manufacturer’s instructions (Miltenyi Biotec). Flow cytometry was performed using BD LSR Fortessa instrument and data were collected with BD FACSDiva 9.0 software. Data were analyzed using FCS Express 7 software (De Novo Software).

### Fluorescent and florescence lifetime imaging microscopy (FLIM) imaging

Objective tumor and stromal response was monitored in real-time using fluorescence microscopy. Fluorescence images were acquired with an Olympus IX70 inverted microscope using SPOT software (SPOT Imaging, Sterling Heights,MI). Confocal images were obtained using a Leica TCS SP8 (Leica) microscope. Fluorescence lifetime imaging microscopy (FLIM) was performed on a Zeiss LSM 710 (Carl Zeiss, Jena, Germany) microscope using an EC Plan-Neofluar 20x/0.50 N.A. objective (Carl Zeiss, Oberkochen, Germany). NADH was excited by an 80 MHz Titanium:Sapphire Mai Tai Laser (Spectra-Physics) at 740 nm. Individual cells were imaged with a size of 256 × 256 pixels and a scan speed of 25.21 μs/pixel. A total of 50 frames were collected and integrated for each fluorescence lifetime image. The excitation and emission signals were separated by a 690 nm dichroic mirror, and a 460/80 bandpass filter and photomultiplier tube (H7422P-40, Hamamatsu Photonics, Hamamatsu, Japan) were used for detection. Frequency domain FLIM data were acquired by the A320 FastFLIM FLIMbox (ISS, Champaign, IL) and analyzed by SimFCS software (LFD, Irvine, CA, www.lfd.uci.edu).

### Analysis of metabolic patterning from FLIM imaging

Image processing (overlay of spot contours and convex hulls) was done using built-in functions in MATLAB’s Image Processing Toolbox. Briefly, a color channel of an image was converted to a binary image based on a manually chosen threshold dependent on staining intensity. A noise filter was applied to reduce background staining. Thresholds were then chosen to define cutoff values of spot boundaries. Parameters for built-in tools were chosen manually to give the best fit for pattern contours. The convex hulls are used to compute the nearest neighbor distances and the area of each spot in microns. The number of spots refers to number of resulting spots outlined from the image processing, after combining spots if their centroids are within a prescribed distance. Thresholds for image analysis were set by user input to capture strongest signals from the epithelial part of the tumors, avoiding stroma and vessels.

### Image analysis

ImageJ software (National Institutes of Health) was utilized to measure the total fluorescence intensity (ie, mean gray value) for each tumor image to quantify tumor growth. Each chamber was normalized to time 0 baseline. Fluorescent and brightfield images are analyzed with ImageJ software. Images are thresholded and overall fluorescence intensity per area is measured as a read-out of tumor growth. Vessels are routinely perfused with 75 kD FITC-dextran to ensure vascular integrity. Each device serves as its own control normalized to baseline time zero pre-drug. FLIM images were analyzed by the phasor approach as previously described.^16^ Briefly, every pixel of the integrated FLIM image is transformed into a point on the phasor plot. The g and s coordinates in the phasor plot are calculated from the sine and cosine components of the Fourier transform of the fluorescence intensity decay of each pixel in the image. By NADH FLIM phasor analysis, we mapped the free to protein bound NADH distribution in the images, which has been correlated to the metabolic state of the biological sample.^17^

### Microfluidic device fabrication

Device fabrication has been described previously.^13–15^ Briefly, polydimethylsiloxane (PDMS, Sigma-Aldrich) is poured into a customized polyurethane master mold of the VMT device design and allowed to polymerize overnight in a 70°C oven. The PDMS replicate layer is removed and holes are punched to create inlet and outlets for the media reservoirs and loading chambers. A thin PDMS membrane is then bonded to the device using oxygenated plasma cleaner (Harrick Plasma). The high throughput system (HTS) is further attached to a 96-well open-bottom plastic culture plate via chemical gluing and oxygen plasma bonding.

### Loading the microfluidic device

Fresh patient-derived samples (2 × 10^6^ cells/mL), EC and LF (both 7 × 10^6^ cells/mL) were resuspended in fibrinogen (10 mg/mL in EBM-2). The cell slurry was then mixed with 6 U thrombin (3 U/μL) to catalyze gel solidification and quickly loaded into the VMT. Fibrin ECM was allowed to solidify at 37°C for 15 minutes prior to introducing laminin into the microfluidic channels to promote vessel anastomosis. VMT were fed with EGM2 and cultured at 37°C, in a 5% CO_2_/20% O_2_ incubator. Media are changed every other day in the VMT and media flow is restored every day in the HTS.

### Drug treatment

Primary-derived CRC grown in the pVMT or as spheroid cultures were randomized to control and treatment groups. Treatment consisted of FOLFOX (FOLFOX), combination 100 μM 5FU, 5 μM oxaliplatin, and 10 μM leucovorin, or galunisertib 1 μM. Drugs were added at day 3–7 and removed after 48 hours to mimic the *in vivo* pharmacodynamics of each drug *in vitro*, since 5FU is not immediately metabolized or excreted under standard culture conditions.^18^ At 48 hours, manual cell counting was performed on spheroid suspension cultures. Imaging was performed on pVMT every 48 hours for 6 days post-treatment.

### Statistics

The relative percent change in pVMT tumor volume for pVMT assays was calculated under each treatment and grouped into response vs. no response. A repeated measures analysis of variance was performed to compare the percentage change in tumor volume between control and drug groups. Pairwise comparisons were used to compare the mean relative percent change in tumor volume under each drug setting vs. control with a standard cut-off of *P* < 0.05. A Bonferroni correction for pairwise multiple comparisons was applied. Data are represented as mean +/− standard deviation of at least 3 independent experiments derived from biological replicates, except when stated otherwise. Measures of association, including correlation coefficients, will be used to estimate sample-size requirements of future trials aimed at obtaining precise estimates (that is, narrow half-widths of 95- and 99-percent confidence intervals) of the degree to which the pVMT results are predictive of patient outcomes.

## RESULTS

### A. The VMT model supports the growth of patient-derived tumor tissue.

**Fig 1.**
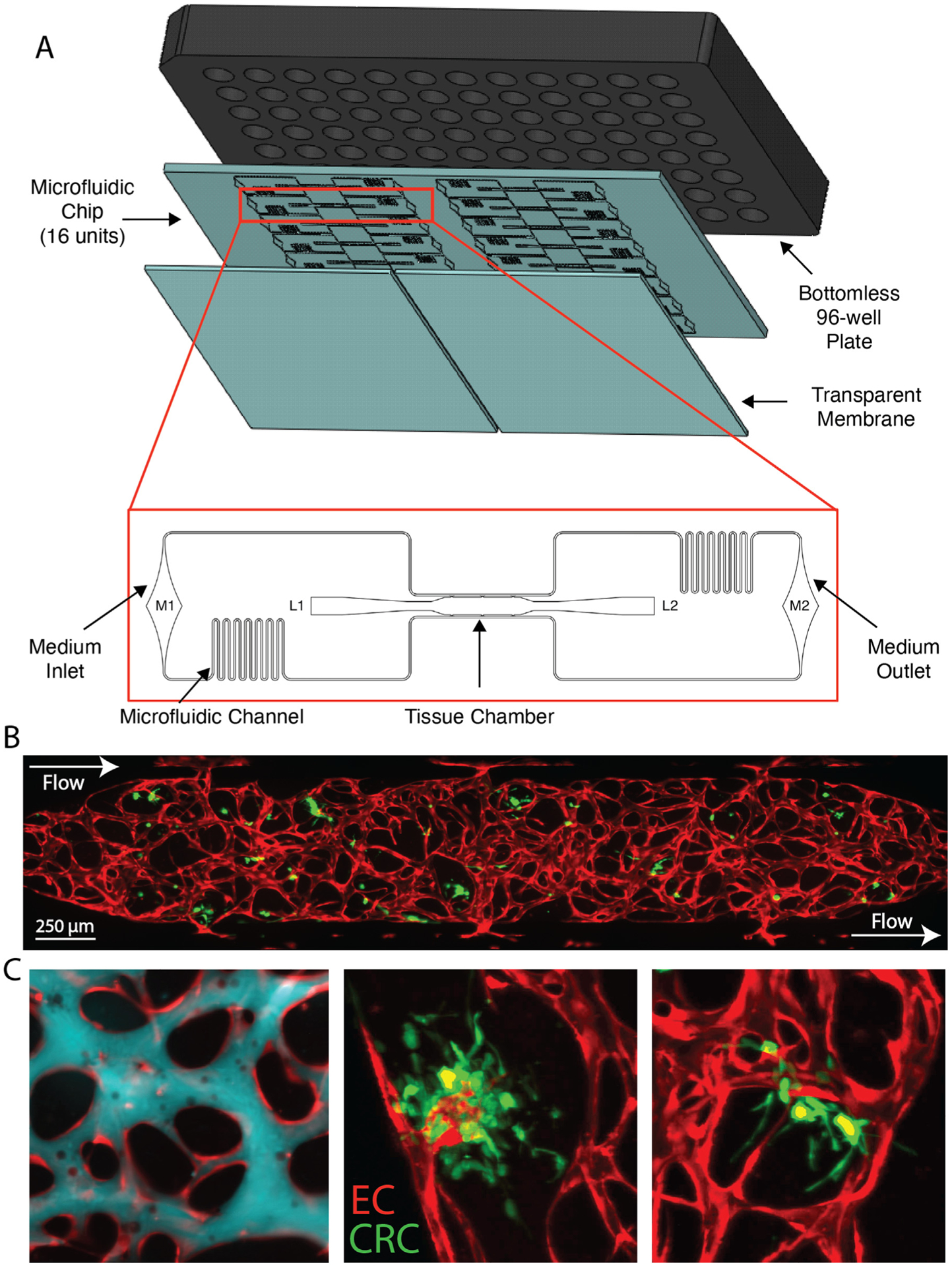
Primary CRC cells grow in a vascularized microtumor model arrayed for high throughput experiments. (**A**) The microfluidic chip is bonded to a bottom-less 96-well plate via chemical glue and oxygen plasma. Zoom view shows a single device unit with a single tissue chamber fed through microfluidic channels, 2 loading ports (L1–2), and medium inlet and outlet (M1–2). (**B**) Fluorescent image of a patient-derived VMT. P54 CRC primary tumor in green, vessels in red. Arrows show direction of flow from high pressure arteriole (top left), through the microvascular bed, out of the low pressure venule (bottom right). (**C**) Far left panel: Vasculature (red) is perfused with 70 kD dextran (blue). Middle and right panels: Zoom views of 2 individual P54 pVMT replicates. As shown, the tumor closely associates and integrates with the vasculature. P54 CRC are expressing green fluorescent protein (GFP).

We have previously validated the VMT model system for cancer-specific disease modeling and drug screening studies using established tumor cell lines.^13–15^ Each single unit chamber is arrayed onto a multiunit chip for high throughput experiments (Fig 1, A). In response to physiological flow, cells self-assemble into microtumors fed through a fully perfused, living vascular network (Fig 1, B). Primary-isolated CRC initiating cells growing within replicate device units integrate with microvessels to form the patient-derived VMT (Fig 1, C) and can be grown for up to 3 weeks. Fully perfused and functional vasculature is present by day 5 at which time tissues can be used for drug screening and other molecular analyses.

To validate the VMT platform as a clinically relevant drug screening model, we obtained fresh, treatment-naive CRC biopsies and surgical samples from eligible and consenting patients during routine, clinically indicated endoscopic or surgical procedures (Fig S1, A). Cancer cells were isolated from primary tissues by gentle dissociation and briefly expanded (~1 week) as nonadherent cultures in serum-free media prior to device loading (Fig S1, A). Human subjects research was carried out in accordance with ethical principles under an IRB-approved protocol. Clinical data (patient demographics, tumor pathology, treatment outcomes, and survival outcomes), when available, were collected retrospectively in compliance with HIPAA regulations. Sixty-three specimens (50 biopsies and 13 surgical resections) were collected to optimize preparation and culture of cells. As shown in Fig S1, B, sample contamination was greatly reduced by the addition of antibiotic-antimycotic, and cell viability was improved dramatically by addition of 10 μM ROCK inhibitor in transport media. Further improvements derived from modifications to tissue digestion, cell maintenance and labeling, and pVMT loading protocols. Specimen biobanking efforts were improved with the collection of surgically resected tumor tissues that provided a greater number of viable cancer cells to perform experiments and cryopreserve. From 26 viable and noncontaminated CRC specimens that were introduced into the VMT, we found that 23 (~88%) successfully grew, and we further performed drug screening on 6 of these patient-derived samples.

In addition to studying these freshly-derived primary CRC cells, we performed drug testing on CCIC, which are patient-derived cells that have been shown to produce tumors resembling the parental tumor when introduced into immunodeficient mice as patient-derived xenografts (PDX).^19^ For the PDX-derived CCIC samples C707 and C1024, mutation status, but not clinical data, is available from Chen et al.^20^ and summarized in Table S1. Tumor biopsy fragments or cancer spheroids were harvested, mixed with endothelial cells (EC) and fibroblasts in extracellular matrix, and introduced into the platform. While we have previously utilized a chip design with 3 chambers per device unit (Fig S2, A), in order to facilitate loading and distribution of tumor spheroids, when used, a single, wide channel design was employed instead (Fig S2, B). Dimensions between the 2 devices are similar, except, to accommodate loading spheroids, the single chamber device has a wider loading channel (0.50 mm compared to 0.20 mm) and contains only 1 compartment (0.10 mm deep).

**Fig 2.**
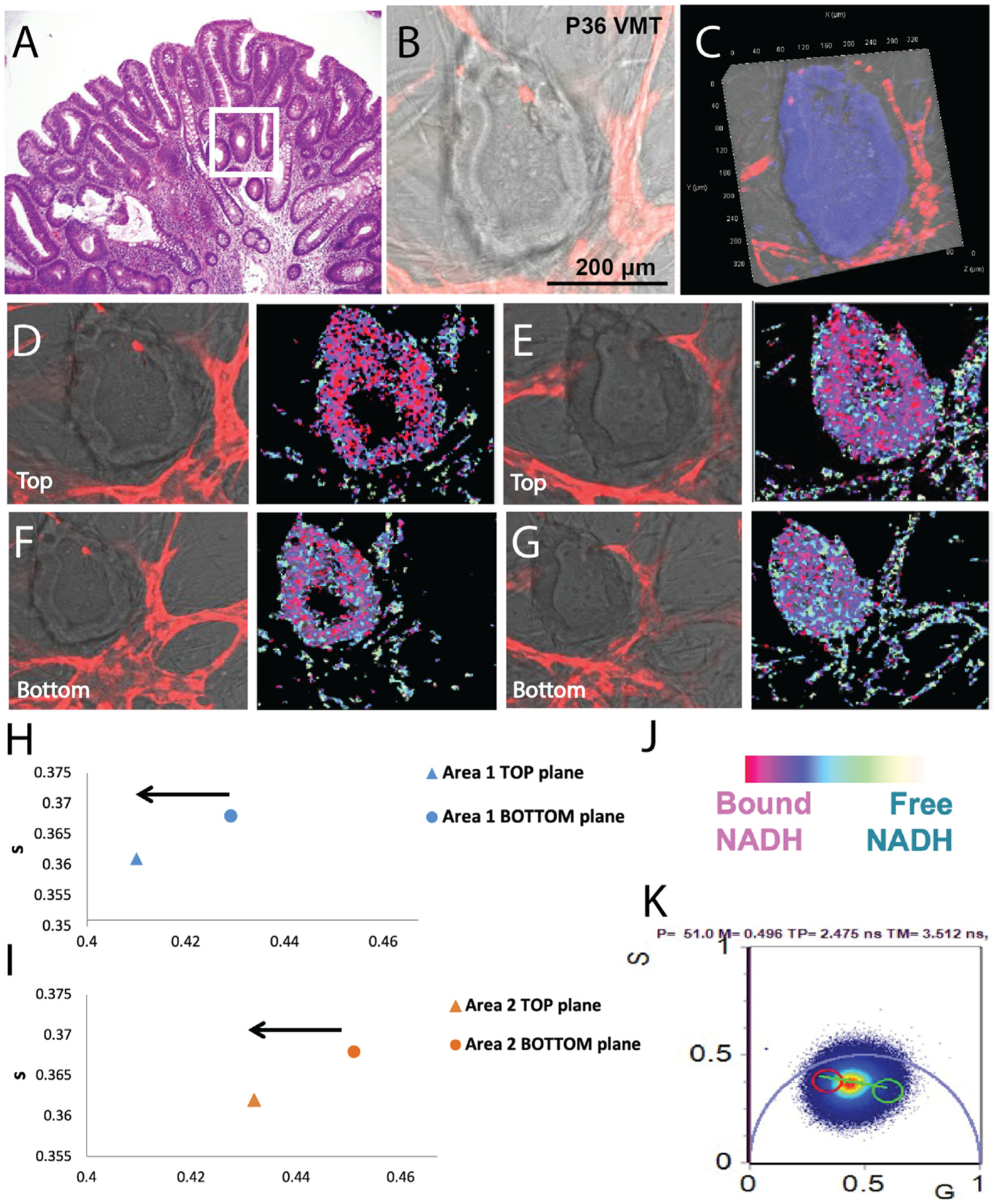
FLIM metabolomics imaging of adenoma-derived pVMT reveals heterogeneity. (**A**) Example H&E stained tubulovillous adenoma. Area of interest shown with white outline is retained in 2B. (**B**) Brightfield/fluorescence merged image of pVMT derived from P36, who had a tubulovillous adenoma. Tumor is unlabeled and vessels are mCherry labeled (red). Shown is a portion of tumor growing within the VMT that recapitulates the parent tumor histology in 2A. (**C**) Brightfield/fluorescence merged image in 2B showing DAPI stain. (**D**) Left: Area 1 top plane merged brightfield/fluorescence images of P36 grown in the pVMT on day 7 of culture with mCherry labeled EC. Right: Resulting metabolomic profile. (**E**) Left: Area 1 bottom plane of P36 with resulting FLIM image on the right. (**F**) Area 2 top and (**G**) bottom planes of P36 with resulting FLIM images. (**H**) Plot of area 1 in top (2D) and bottom (2E) planes showing metabolic shift. (**I**) Plot of area 2 in top (2F) and bottom (2G) planes showing metabolic shift. (**J**) Color legend of FLIM images. (**K**) Representative phasor plot.

The VMT robustly supports the survival and growth of patient-derived CRC tumors from different individuals (Fig S3). Via co-culture of multiple cell types within an extra-cellular matrix, a dynamic tumor microenvironment is formed within the pVMTs to support primary CRC tumor growth from various cancer stages. In Fig. 2, A, a hematoxylin and eosin (H&E) stained slide of a tubulovillous adenoma shows typical histological features that are recapitulated in pVMT established from primary sample P36 (Fig 2, B). P36 is derived from a rectal tubulovillous adenoma with high-grade dysplasia that grows in the pVMT. Importantly, we have found that the pVMT readily supports tumors derived from not only later stage tumors but also early stage disease and premalignant lesions, for which successful growth has proven to be a major challenge in current culture systems and mouse models.^7, 8, 21, 22^ The growth of precancerous polyps within the pVMT has profound implications for CRC treatment and prevention by facilitating the study of neoplastic transformation *in vitro*.

### B. FLIM detects metabolomic heterogeneity in pVMT.

Cancer cells often utilize glycolysis to fulfill their metabolic and energy demands rather than oxidative phosphorylation (OxPhos), regardless of oxygen availability, a phenomenon known as the Warburg effect.^23^ Targeting cancer cell metabolism has emerged as a promising therapeutic approach in several solid tumors, including CRC.^24–26^ To visualize cells with high glycolytic activity both *in vitro* and *in vivo*, fluorescence lifetime imaging microscopy (FLIM) can be used as a label-free and noninvasive method.^27, 28^ FLIM measures metabolic activity in living cells using differences in the fluorescence lifetime of free vs. protein-bound NAD(P)H, the key cofactor in many metabolic pathways.^27, 28^ Free NADH correlates with a more glycolytic environment, while the bound form correlates with less glycolysis. FLIM reveals glycolytic gradients throughout the tissue by measuring distinct lifetimes for the free- and protein-bound conformations of NAD(P)H, thereby providing a metabolic signature.^28^ We have shown this method to be highly sensitive in distinguishing the metabolic state between different cell types in the VMT.^13^ In Fig S4, FLIM imaging shows an SW480 CRC tumor growing within the VMT, with areas of highly glycolytic “hot-spots” adjacent to areas with low glycolytic activity. Nearest-neighbor analysis of these highly metabolic regions in 3 independent SW480 VMT tumors shows strong overlap with similarly analyzed SW480 tumors *in vivo*, suggesting similar metabolic patterning in the VMT and *in vivo* tumors.

**Fig 3.**
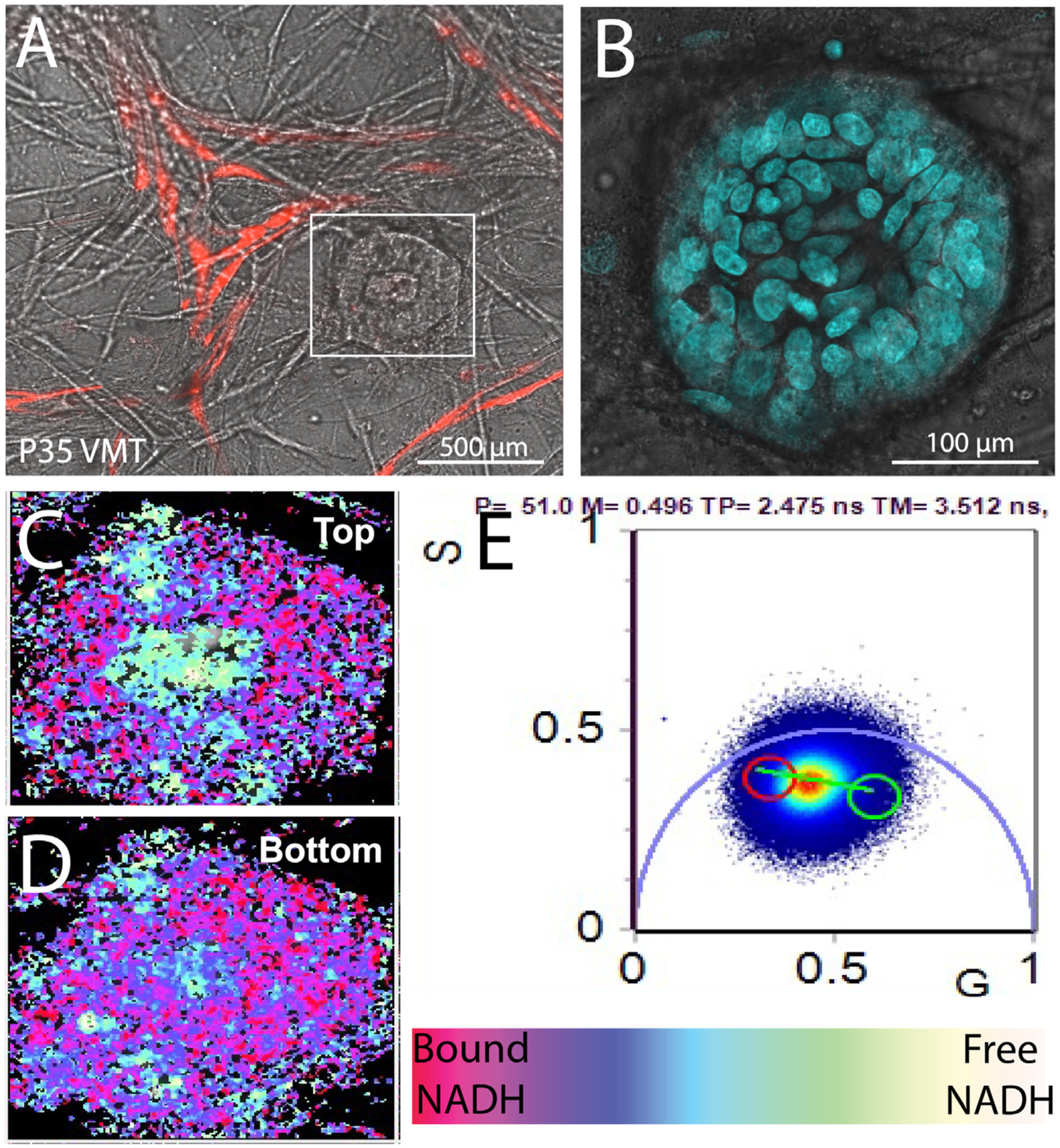
FLIM metabolomics imaging of stage I rectal cancer-derived pVMT reveals heterogeneity. (**A**) P35 growing in the pVMT with vessels labeled with GFP. (**B**) Confocal image showing DAPI staining of P35 in the pVMT. (**C**) Resulting FLIM image of top plane of P35. **(D)** Resulting FLIM image of bottom plane of P35. (**E**) Phasor plot with color legend.

Intriguingly, high-resolution FLIM z-stack imaging of P36 (Fig 2, D–G) shows a high degree of spatially patterned metabolic heterogeneity in a top-down pattern that models patterns observed in colon crypts *in vivo*,^17^ demonstrating tissue-specific metabolic patterning within the pVMT microenvironment. Quantification of metabolic shift between the top and bottom planes for 2 distinct tissue areas is shown in Fig 2, H and I, with color legend for FLIM images (Fig 2, J) and representative phasor plot (Fig 2, K). Briefly, each pixel of the integrated FLIM image is transformed into a point on the phasor plot and the g and s coordinates in the phasor plot are calculated from the sine and cosine components of the Fourier transform of the fluorescence intensity decay of each pixel in the image. By NADH FLIM phasor analysis, we mapped the free to protein bound NADH distribution in the images, which shows a distinct shift in the metabolic state across different areas of the patient-derived sample. For the rectal stage I tumor P35 grown within the pVMT (Fig 3, A), DAPI staining confirms a solid tumor mass (Fig 3, B). FLIM imaging of different planes of the tumor reveals metabolic heterogeneity (Fig 3, C and D), with more glycolytic activity in the core of the tumor mass and distinct areas of high free (NADH) interspersed with high bound NADH similar to what has been observed in solid tumors *in vivo*.^17^ A representative phasor plot is shown in (Fig 3, E). Our findings demonstrate that cells in the pVMTs mimic *in vivo* metabolomic patterning.

### C. Primary CRC derived from different patients represent heterogeneous disease pathologies.

From 4 patients with CRC, we prospectively derived stable cultures of primary cancer cells for drug sensitivity testing within pVMTs and spheroid cultures. The patient characteristics and tumor sites are shown in Table III, with all samples collected (randomly) from male patients. Demographics included White/non-Hispanic (n = 3) and Asian/non-Hispanic (n = 3). Ages ranged from 67 to 86 years old (median age: 75), and tumor site included colon (n = 3) and rectum (n = 1).

H&E stained FFPE (formalin-fixed, paraffin-embedded) tissue sections from each primary-derived tumor specimen (P54, P59, P61, and P63) in each case reveals heterogeneous histology (Fig S5, A), from which EpCAM+ epithelial cells were positively selected for *in vitro* in the absence of factors known to be required for normal epithelial cell growth^12^ and subsequently loaded into the VMT platform (Fig S5, B and C). Primary CRC specimens were derived from adenocarcinomas with varied pathological characteristics and TNM staging (Table III), with the majority (n = 3) coming from stage II disease with no lymph node involvement. One sample, P61, was obtained from a patient with stage III CRC and 3 affected lymph nodes. Tumor grade was similar between the patient specimens. The majority of samples (n = 3) were derived from moderately differentiated tumors, with the exception of specimen P63, which was derived from a moderately to poorly differentiated tumor. The microsatellite instability (MSI) status of all 4 samples was determined during clinical pathological evaluation, and tumors were found to be microsatellite stable (MSS) subtype.

**Fig 4.**
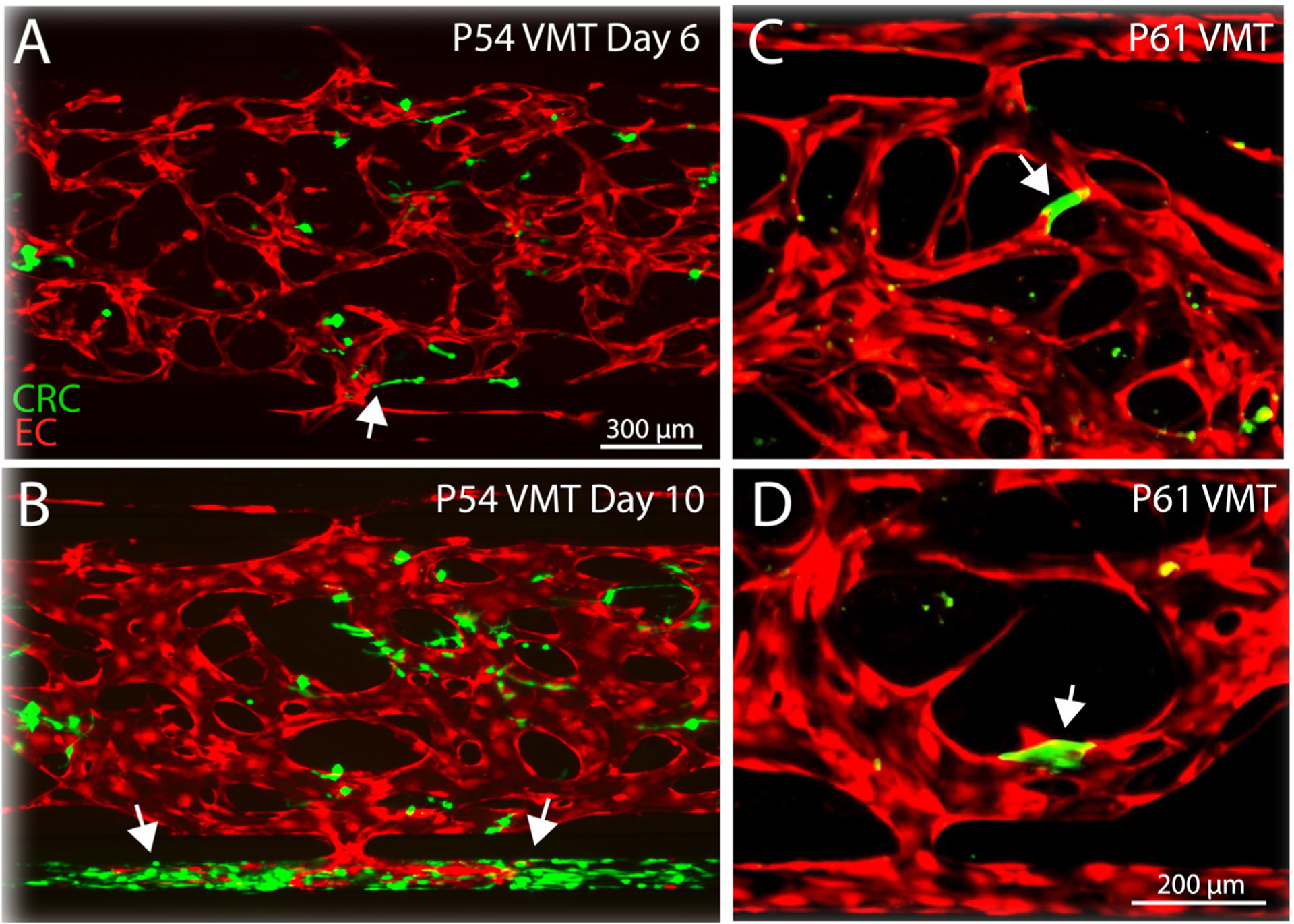
pVMTs derived from P54 and P61 specimens display growth characteristics of invasive CRC disease. (**A**) Fluorescence image shows GFP labeled P54 CRC cells grown in the pVMT with mCherry labeled EC on day 6. Arrowhead shows area where P54 cancer cells are elongating toward the vasculature to intravasate. (**B**) Fluorescence image shows GFP labeled P54 pVMT from (**A**) on day 10. Arrowheads show areas where P54 cancer cells have extravasated from the vasculature into the outer channel and rapidly proliferated. (**C**) and (**D**) Magnified views of GFP labeled P61 CRC cells demonstrating vascular mimicry within the pVMT by associating with mCherry labeled EC to form a continuous vessel.

Patient-derived tumor P54 is the only sample to have next generation sequencing (NGS) performed in clinic, and was found to harbor 5 mutations per megabase of DNA, which is close to the median for CRC.^29^ Sequencing revealed a KRAS G12V mutation but wild-type BRAF and NRAS, APC I1307K mutation, TP53 R175H mutation, and amplification of ERBB2, CCND2, FGF23, FGF6, and KDM5A. Intriguingly, the pathology report for P54 tumor specimen notes focal vascular invasion, which we see recapitulated only in pVMT derived from P54 (Fig 4, A and B). In contrast to the morphology of P54 CRC cells grown in spheroid cultures, the pVMT fosters a microenvironment where the cancer cells readily associate with the nearby vasculature and adopt an invasive, elongated phenotype (Fig 4, A). The P54 CRC cells grow and invade nearby vessels, gaining access to the outer channels and proliferating rapidly (Fig 4, B), likely in response to nutrient delivery through the perfused vasculature, mimicking the pathological behavior of the parental tumor.

The patient who donated P61 had stage III disease at the time of CRC resection, with an overall survival of 20 months (Table III). For P61, we observe a striking cancer cell phenotype within the pVMT that resembles vascular mimicry (Fig 4 C and D). Vascular mimicry occurs when aggressive tumor cells mimic vasculogenic networks as a supplement to blood vessels to maintain tumor growth and metastasis.^30, 31^ A recent study assessing vascular mimicry in CRC found that vascular mimicry is prognostic of decreased survival.^32^ As shown in Fig 4, C and D, a subset of GFP-labeled P61 cancer cells readily incorporate into the pVMT vasculature, replacing EC in portions of the vascular network. Together, these findings demonstrate that the pVMT can capture clinical pathological findings and cellular behaviors not readily observable in standard model systems.

### D. Primary CRC in the pVMT show heterogeneous sensitivities to standard-of-care chemotherapy and TGF-*β*R1 inhibition in the pVMT.

We next sought to determine the sensitivity of each primary-derived CRC sample to a standard chemotherapeutic regimen of FOLFOX, which is standard first-line treatment for CRC. Since we have previously tested galunisertib, a potent TGF-*β*R1 inhibitor, and found it to be highly efficacious in SW480 VMT, we also treated pVMT with galunisertib to determine if TGF-*β*R1 warrants further investigation as a novel target in CRC. Treatment duration was based on previous studies comparing VMT response to FOLFOX for 2 CRC cell lines, SW480 and HCT116, with those same tumors xenografted into mice.^15^ Strikingly, VMT and *in vivo* tumor growth and response tracked closely during the 1-week assessment period postchemotherapeutic treatment.

**Fig 5.**
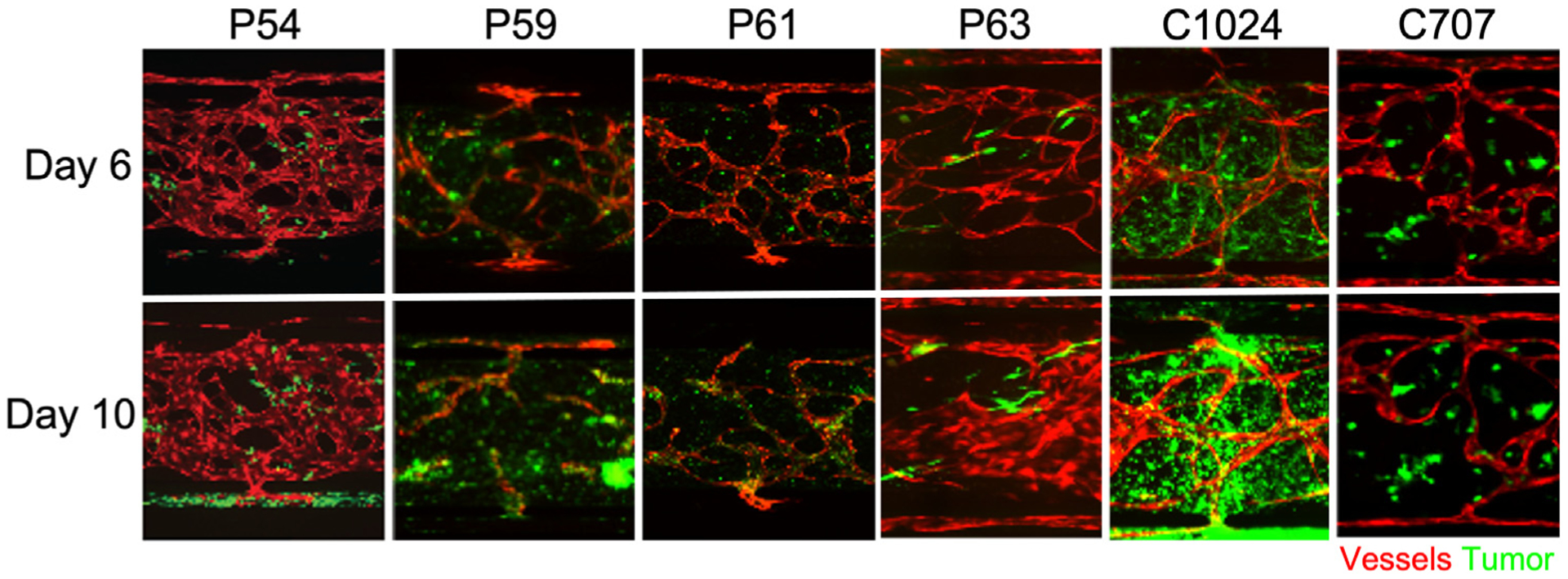
The VMT supports the survival and growth of patient-derived CRC cells from different individuals. Fluorescent merged images of pVMT. Patient-derived CRC cells (Venus, green fluorescence) are seeded into each device unit with EC (mCherry, red fluorescence) and fibroblasts (unlabeled). Shown are 4 primary CRC tumors derived from different individuals (P54, P59, P61, and P63) and 2 PDX-derived lines (C1024 and C707). The top row shows the pVMT on day 6 of culture and the bottom row shows the same pVMT on day 10 of culture.

**Fig 6.**
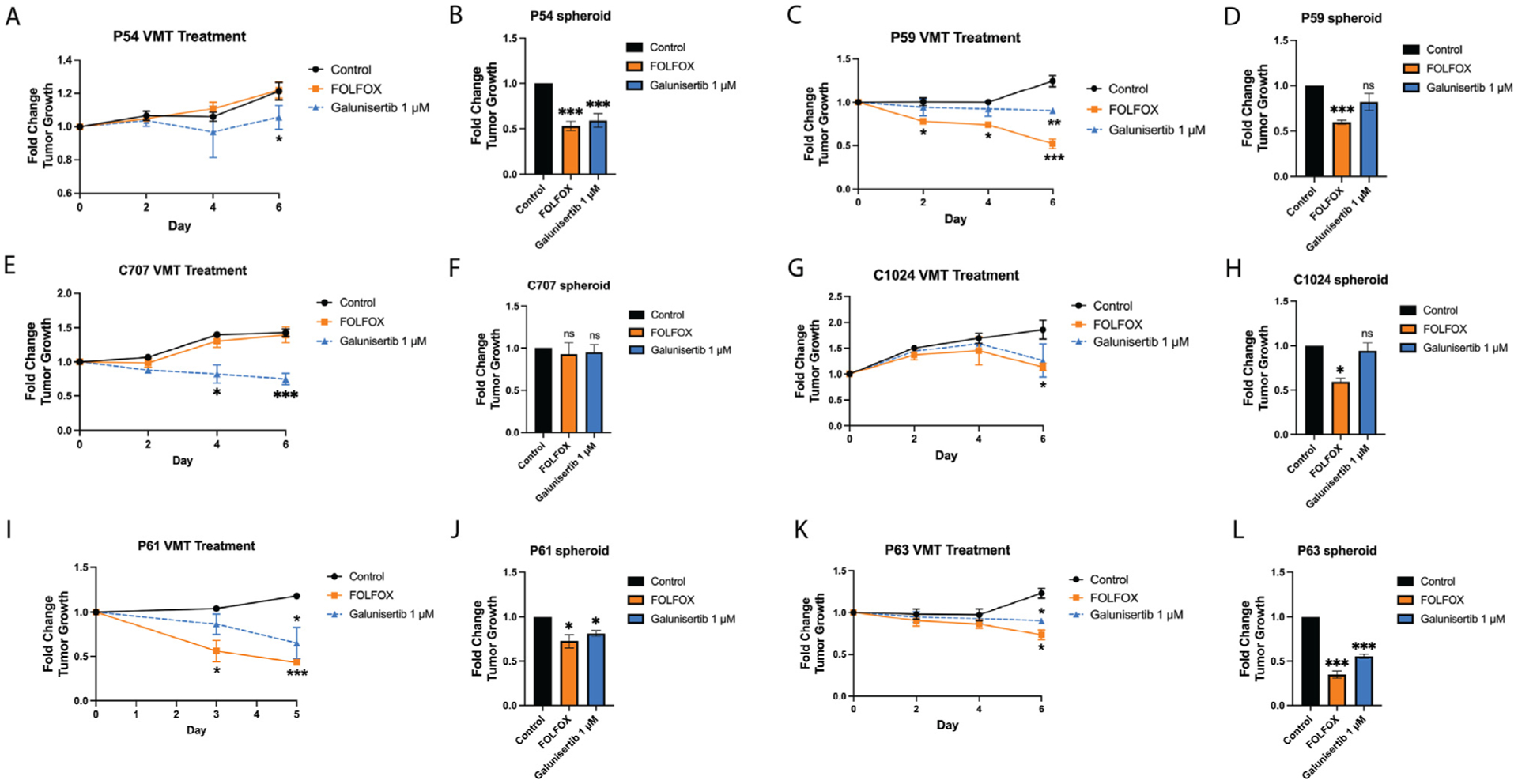
pVMT demonstrate heterogeneity in drug sensitivities between primary samples within the VMT and in contrast to spheroid cultures. (**A**) Quantification of P54 VMT treatment. (**B**) Quantification of P54 spheroid treatment. (**C**) Quantification of P59 VMT treatment. (**D**) Quantification of P59 spheroid treatment. (**E**) Quantification of C707 VMT treatment. (**F**) Quantification of C707 spheroid treatment. (**G**) Quantification of C1024 VMT treatment. (**H**) Quantification of C1024 spheroid treatment. (**I**) Quantification of P61 VMT treatment. (**J**) Quantification of P61 spheroid treatment. (**K**) Quantification of P63 VMT treatment. (**L**) Quantification of P63 spheroid treatment. * p < 0.05; ** p < 0.01; *** p < 0.001; ns = non-significant.

We compared treatment responses in the pVMT with responses in standard spheroid cultures to determine the degree to which the tumor microenvironment alters drug sensitivity. Representative fluorescent micrographs of each pVMT from day 6 and day 10 of culture are shown in Fig 5. The results for pVMT and spheroid drug screening are shown in Fig 6 and Table IV.

Interestingly, P54 CRC cells, which demonstrate metastatic behavior, are completely resistant to FOLFOX treatment within the pVMT, showing no sensitivity to the chemotherapy even 6-days post-treatment initiation (Fig 6, A). This is in stark contrast to the response observed in spheroid cultures, where FOLFOX treatment significantly reduced tumor growth (~50%) after only 2 days of treatment (Fig 6, B). The treatment response to galunisertib was also attenuated in the pVMT compared to spheroid culture, with a slight but significant decrease (~20%) in tumor growth observed at the latest time point in the pVMT (Fig 6, A). Galunisertib shows a greater effect in spheroid culture with approximately 50% reduction in tumor growth 2 days post-treatment (Fig 6, B). Clinically, the patient who donated P54 initially declined chemotherapy after surgical CRC resection (stage II). At progression 9 months later, he did begin combination treatment with capecitabine, which is an oral prodrug of 5-FU, and bevacizumab, a humanized monoclonal antibody specific for VEGFA (Table III). He declined oxaliplatin due to toxicity. The total duration of bevacizumab treatment was 6 months, at which point he progressed and was then treated with radiation therapy for 1 cycle. His overall survival was 20 months (Table III).

In contrast to P54, primary CRC sample P59 shows a high degree of sensitivity to FOLFOX (5FU, oxaliplatin and leucovorin) treatment in the pVMT, with significant response (~25% reduction in tumor growth) at 2 days and 4 days post-treatment, and ~50% regression by day 6 (Fig 6, C). Consistent with the pVMT data, P59 spheroid cultures treated with FOLFOX showed a ~50% decrease in CRC cell growth at 2 days post-treatment (Fig 6, D). The patient who donated P59 tumor did receive treatment after surgery with 4 cycles of capecitabine plus oxaliplatin (CAPOX), followed by 4 cycles of capecitabine (Table III). He was last seen at follow-up with no evidence of disease, indicating that his surgery and treatment were highly efficacious. P59 CRC cells also show sensitivity to galunisertib at day 6 post-treatment in the pVMT, with a significant effect of approximately 25% reduction in tumor growth (Fig 6, C). Intriguingly, the effect of galunisertib on P59 is only observed in the pVMT, and not in spheroid cultures (Fig 6, D), suggesting a potential role for the tumor-associated stroma in treatment response. For CCIC line C707 (Fig 6, E), the difference in effect size for galunisertib is even more striking. While C707 shows no response to FOLFOX treatment in the VMT, there is a significant 40% reduction in tumor growth in response to galunisertib at 4 days post-treatment, that increases to 60% regression at 6 days post-treatment (Fig 6, E). The C707 spheroid cultures recapitulate the FOLFOX insensitivity seen in the VMT (Fig 6, F), but completely miss galunisertib as a potential targeted therapy option — the drug having no effect at all in this geometry. Notably, C707 harbors a SMAD4 mutation (Table S1), further supporting a cancer cell extrinsic target that leads to tumor regression in response to TGF-*β*R1 inhibition.^15, 33, 34^ We next assessed drug response for CCIC line C1024, and find that within the VMT, C1024 is resistant to both FOLFOX and galunisertib until 6 days post-treatment, when both therapies result in about 50% regression in C1024 tumors (Fig 6, G). Conversely, C1024 spheroid cultures show significant sensitivity to FOLFOX at 2 days post-treatment with approximately 50% reduction in tumor growth, whereas galunisertib shows no treatment response in C1024 spheroids (Fig 6, H). For P61, sensitivity in the pVMT roughly tracks with response in spheroid cultures (Fig 6, I and J), although the response in the pVMT is attenuated for galunisertib until about day 5. P61 shows a high degree of sensitivity to FOLFOX, with greater than 50% reduction in tumor growth in the pVMT by day 3. Interestingly, we see a significant response for P63 in spheroid cultures at 48 hours treatment with FOLFOX and galunisertib, but response to both regimens is attenuated in the pVMT, with significant targeting of the tumor only seen at day 6 post-treatment initiation (Fig 6, k and L). For both P61 and P63 specimens, we are unable to compare pVMT and spheroid treatment responses with clinical response since these patients did not receive systemic treatment after surgical resection (Table III). Patient P61 was offered adjuvant chemotherapy but declined due to concerns about toxicity.

## DISCUSSION

By optimizing culture conditions to grow patient-derived tumor samples in the VMT for *ex vivo* CRC disease modeling and drug testing, we have developed a truly personal therapeutic screening methodology with potential to improve patient outcomes. Here we show that we can reliably establish patient-derived VMT from both colon and rectal tumors of various stage and grade, and that pVMT growth and response to treatment is heterogeneous. Metabolic heterogeneity arises within the pVMT, showing tumor areas with high free NADH at the tumor core that replicates the metabolomics of *in vivo* tumors. We find that the pVMT supports the growth of a high-grade tubulovillous adenoma (P36), which retains the histology of the parental polyp and demonstrates a top-down metabolic tissue pattern resembling an *in vivo* crypt^17^ as it grows within the pVMT. Stem cells at the base of intestinal crypts have been found to be highly glycolytic and a metabolic trajectory of glycolysis-to-oxidative phosphorylation tracks with a gradient of strong-to-weak Wnt signaling,^17^ suggesting that metabolic patterning in premalignant lesions warrants further investigation.

Strikingly, primary CRC tissue derived from patient P54 display metastatic behavior within the pVMT, mimicking the focal vascular invasion of the parental tumor. Intriguingly, P54 spheroids showed a high degree of sensitivity to FOLFOX, whereas P54 CRC cells growing in the pVMT were completely resistant to FOLFOX treatment. Patient P54 received capecitabine- bevacizumab combination therapy at the time of his progression 9 months after surgery and declined oxaliplatin treatment due to toxicity. He then received bevacizumab alone for 6 months, before receiving 1 cycle of radiation therapy and subsequently declining further treatment. His overall survival was 20 months, indicating that he progressed soon after his treatment with capecitabine, an oral prodrug of 5FU. Only the pVMT, and not the spheroid cultures, derived from his tumor predicted resistance to FOLFOX, a standard chemotherapeutic regimen that includes 5FU and oxaliplatin.

Although patient P54 did not receive FOLFOX, he did receive 5FU, which is a component of FOLFOX, and given that the pVMT better recapitulated P54 tumor pathological behavior *in vitro* than the spheroid cultures, it is likely that the pVMT also better captures novel drug sensitivities of P54. Our findings have critical implications in the choice of preclinical model to inform personalized medicine, since P54 FOLFOX resistance was only detected in the pVMT and not in spheroid cultures. P54 showed some sensitivity to galunisertib treatment in the pVMT, with a greater effect observed in the spheroid cultures. The pVMT detected P54 CRC cell sensitivity to the targeted therapy galunisertib but not FOLFOX, highlighting a case where clinical management may have been improved with information on a druggable target.

As expected based on the high degree of intertumor heterogeneity observed in CRC, we find heterogeneous drug response to TGF-*β*R1 targeted therapy and FOLFOX between patients. The patient who donated P59 tumor tissue received treatment with 4 cycles of CAPOX followed by 4 cycles of capecitabine, presenting with no evidence of disease at last follow-up. A high degree of FOLFOX treatment efficacy was observed for P59-derived pVMT, with ~50% tumor regression observed in the pVMT at day 6 post-treatment. P59 spheroid cultures also showed ~50% reduction in tumor growth by day 2 post-treatment, revealing, in contrast to P54, consistency with the pVMT results and clinical response. However, P59 and most other primary-derived CRC tested in this study show attenuated response to FOLFOX in the pVMT, whereas spheroid cultures show significantly enhanced responsiveness to drug treatment. For P59 and C707, pVMT detected tumor sensitivity to treatment with galunisertib, but spheroid cultures did not. Thus, spheroid cultures may erroneously demonstrate drug efficacy when the actual tumor is resistant and, conversely, may also miss potentially promising therapeutic candidates altogether.

C707 harbors a SMAD4 mutation that renders the tumor cells insensitive to TGF-*β* signaling pathway disruption, lending credence to the hypothesis that the effect of galunisertib treatment we observe on the tumor is through the tumor-associated stroma. We have previously found that SW480 CRC cells, which have mutant SMAD4, are also sensitive to galunisertib in the VMT but not in spheroid cultures, and that SW480 reprogram the fibroblasts within the VMT toward a CAF phenotype with activated TGF-*β* signaling.^15^ A similar phenomenon has been described in clinical CRC disease,^33^ warranting further investigation into the mechanism of action for galunisertib to better understand this pathology. Galunisertib completed Phase II clinical trials for hepatocellular cancer (HCC),^35^ showing minimal side effects, before development was discontinued by Eli Lilly. However, the drug could potentially be re-purposed for another cancer indication, such as CRC, or for a subset of patients with a specific biomarker, if preclinical data support it.

Patient P61 presented with stage III CRC but declined chemotherapy, so we are unable to compare clinical response with the drug sensitivities of the pVMT and spheroids. However, if the patient who donated P61 tumor tissue had undergone adjuvant treatment, it is likely the patient would have received FOLFOX, the standard first-line therapy for MSS CRC, for which both the pVMT and spheroids derived from his tumor predicted sensitivity. Importantly, we observed tumor heterogeneity within the pVMT that was not captured by the spheroid cultures, and a subset of P61 cancer cells displayed vascular mimicry in the pVMT. In the future, we envision assessing vascular mimicry and metastatic behavior in the pVMT, as demonstrated in this study, to guide clinical decisions and inform prognosis. Our findings further underscore the need for individualized drug screening in a physiologically relevant *in vitro* model to guide personalized medicine in oncology.

Based on our results, one can envision testing other drugs and combination therapies in the pVMT to rapidly inform therapy choice for each individual patient. To date, we have shown that the VMT is a powerful tool for screening antiangiogenic agents, small molecule and tyrosine kinase inhibitors, and chemotherapy drugs.^13–15^ We have previously demonstrated the utility of the VMT for testing the efficacy of biologics, including cetuximab and pembrolizumab, and we are also integrating immune components, for example showing tumor-specific killing by perfused T cells (*unpublished results*). Future VMT models will include a hepatic tissue compartment to better mimic drug metabolism. To further personalized medicine efforts, additional tissue compartments may be integrated with the VMT to study the metastatic process and recapitulate *in vivo* pharmacokinetics/pharmacodynamics on-chip.^36^

Although growing cancer cells from clinical tissue is notoriously unpredictable, VMTs replicate the *in vivo* tumor microenvironment more closely than standard monolayer or spheroid cultures and, as such, foster higher rates of successful growth. We see nearly 90% success rate for engraftment of primary-derived tumors in our system (23 tumors out of 26 viable, noncontaminated tumors collected). In contrast, in immunodeficient mice, engraftment success typically ranges from 10% to 50% depending on tumor type and often requires months to establish.^21^ Notably, the VMT also supports successful growth for early-stage disease tissue and premalignant polyps for which primary cultures are otherwise unattainable, allowing possibilities for therapeutic development, testing, and disease modeling for lesions that have not yet progressed.

Additional advantages to the pVMT are that it can be established within days of tissue collection and it is much easier to monitor and manipulate than xenograft models, allowing for results to be acquired and interpreted in near real-time. Furthermore, the pVMT incorporates only human cells and requires very few cells for establishment, such that drug testing can be performed in a rapid and high throughput manner using tumor cells derived from a small biopsy. Although it takes 5 days to establish the current pVMT version, future iterations will utilize a dual-chamber microfluidic device design that allows perfused vasculature to form in advance and separately from the tumor, facilitating immediate drug testing at the time of tumor collection. Based on our findings, we conclude that the pVMT serves as a clinically relevant model for drug screening and personalized medicine applications.

## CONCLUSION

The need for improved therapies in CRC is immense. Despite the large global burden and poor prognosis, our molecular understanding of CRC and ability to predict response to therapy has been slow to translate to clinical benefit. Although recent molecular insights have begun to pave the way for disease stratification and development of targeted cancer therapies for improved patient outcomes, the full potential of current research efforts has yet to be realized. Improving the preclinical prediction efficacy of human drug responses and stratifying patients in clinical trials based on molecular profile are both critical to reducing costly failures in drug development and to ensuring that each patient receives the optimal treatment regimen tailored to their disease given malignancy. Toward this goal, we have optimized culture conditions to grow patient-derived tumor samples in the VMT and show that the pVMT is a clinically relevant physiologic *in vitro* model for the study of CRC. We envision that results from the pVMT can be used to guide clinical decisions, complementary to current molecular-based approaches including ctDNA analyses. Notably, we have isolated tumor-derived exosomes from VMT effluent (*unpublished data*), therefore we also anticipate that the pVMT will be useful as a tool to validate clinically relevant biomarkers. In future studies, we plan to use the pVMT model to better understand the role of TGF-*β* signaling in CRC tumor biology and to study different stages of tumor progression and dissemination. Our goal is to facilitate development of novel therapeutic approaches tailored to individual patients, and, ultimately, to accelerate translation of the pVMT as a diagnostic tool to guide clinical management. This new approach to personalized medicine is necessary to ensure that the most clinical benefit, with the least toxicity, is consistently achieved from therapy for each individual patient.

## Supplementary Material

S2

S1

S3

S4

S5

## Figures and Tables

**Table I:** CCIC basal media

Reagent	[Original]	[Final]	Volume for 50 mL
DMEM F12 50:50 media	n/a	n/a	48 mL
Nonessential amino acids	100× (10 μM)	1× (0.1 μM)	500 μL
Pen-strep	100×	1×	500 μL
B27 supplement	50×	0.2×	200 μL
Heparin	2 μg/μL	4 μg/mL	100 μL
Sodium pyruvate	100×	1×	500 μL

**Table II:** CCIC complete media components

Reagent	[Original]	[Final]	Volume for 50 mL
DMEM F12 supplemented as above	n/a	n/a	50 μL
bFGF	100 ng/μL	20 ng/mL	10 μL
EGF	0.5 pg/μL	40 ng/mL	4 μL
N2	100×	1×	500 μL

**Table III:** Patient treatments and survival outcomes

Patient number	Age	Gender	Race/ethnicity	Tumor site	TNM	Tumor stage	LN’s	Grade	Treatment	PFS	OS	Progression to metastatic?
P54	81	M	White / non-Hispanic	Rectum	T3N0M0	II	0/17	Moderate	Surgery (abdominal perineal resection) then capecitabine-bevacizumab, bevacizumab alone, radiation	9 months	20 months	Yes
P59	69	M	Asian / non-Hispanic	Colon (Cecum)	T4N0M0	II	0/20	Moderate	Surgery with adjuvant CAPOX x 4 cycles, then capecitabine x 4 cycles	n/a	NR at 32 months	No
P61	86	M	White / non-Hispanic	Descending Colon, Sigmoid Colon	T4N1M0, T3N1M0	III	3/23, 3/23	Moderate	Surgery (Colectomy)	n/a	20 months	No
P63	67	M	White / non-Hispanic	Colon (Sigmoid)	T3N0M0	II	0/16	Moderate to poor	Surgery (sigmoidectomy)	n/a	NR at 22 months	No

**Table IV:** Response of each primary CRC to FOLFOX and galunisertib

Primary CRC	Response to FOLFOX			Response to galunisertib	
	Clinic	Spheroid	VMT	Spheroid	VMT
P54	No	Yes	No	No	Yes
P59	Yes	Yes	Yes	No	Yes
P61	-	Yes	Yes	Yes	Yes
P63	-	Yes	Yes	Yes	Yes
Cl024	-	Yes	Yes	No	Yes
C707	-	No	No	No	Yes

## At A Glance Commentary

Hachey et al.

### Background

A major obstacle to personalized cancer treatment is the lack of technologies that can identify drug sensitivity for each individual patient. Herein we describe a vascularized microtumor (VMT) model system for drug screening that recapitulates the complex, heterogeneous microenvironment of a human tumor *in vitro*.

### Translational Significance

We show that the VMT reproducibly supports the long-term culture of patient-specific tumor cells derived from small fresh CRC biopsies, and accurately models tumor growth, heterogeneity, and drug response seen in clinical specimens. As such, the VMT may serve as a powerful tool to accelerate translation of personalized treatments to the clinic.
