# Supplementary figures and images for: A human vascularized microtumor model of patient-derived colorectal cancer recapitulates clinical disease
Source: Transl Res. Author manuscript; Available in PMC 2023 Oct 23. (PMC10593408; doi:10.1016/j.trsl.2022.11.011)

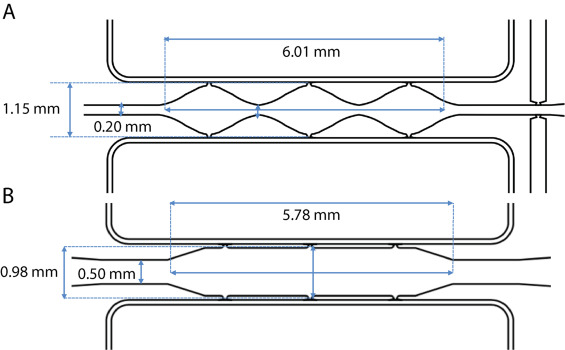

Supplement: S2 [file NIHMS1930003-supplement-S2.jpg]

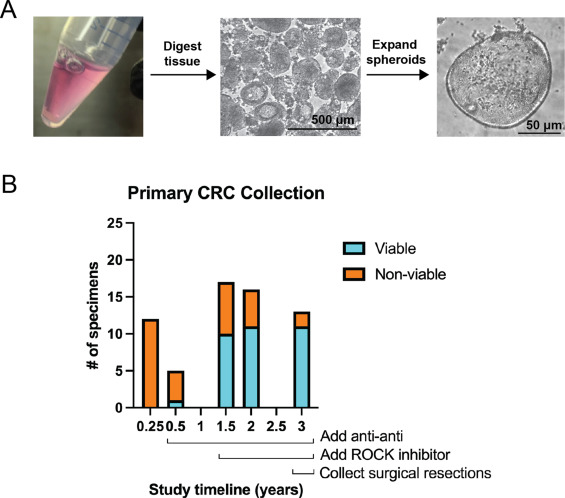

Supplement: S1 [file NIHMS1930003-supplement-S1.jpg]

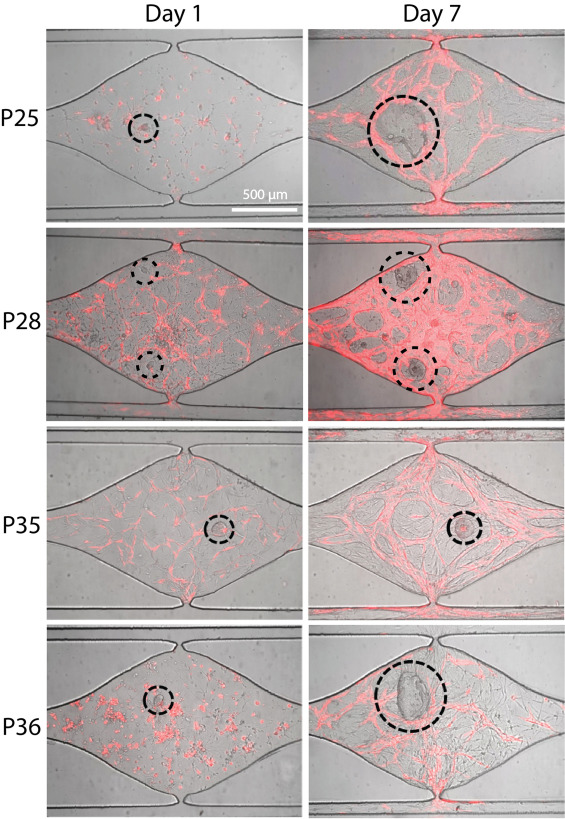

Supplement: S3 [file NIHMS1930003-supplement-S3.jpg]

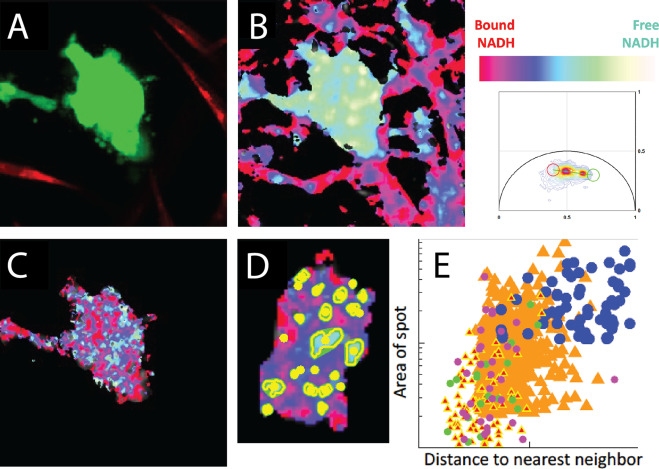

Supplement: S4 [file NIHMS1930003-supplement-S4.jpg]

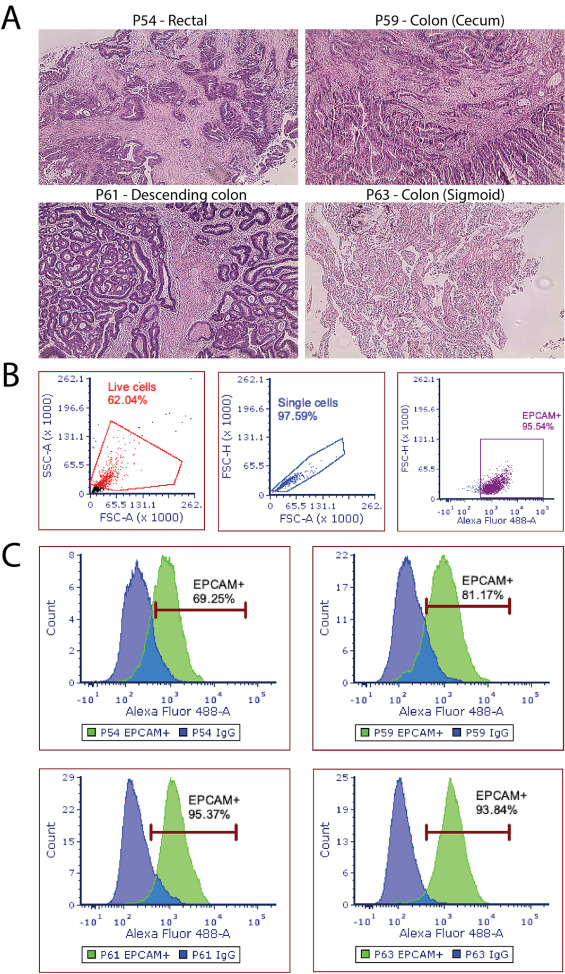

Supplement: S5 [file NIHMS1930003-supplement-S5.jpg]
